# Hepatic clamping and postoperative hyperglycaemia: A retrospective cohort study in liver surgery

**DOI:** 10.1097/EA9.0000000000000123

**Published:** 2026-06-24

**Authors:** Gabriel Thierry, Florian Beck, Olivier Jacquemin, Pierre-Yves Hardy, Abdourahmane Kaba, Morgan Vandermeulen, Olivier Detry, Vincent Bonhomme

**Affiliations:** From the Department of Anesthesia and Intensive Care Medicine, Liège University Hospital, Liège, Belgium (GT, FB, OJ, PYH, AK, VB), Groupe francophone de Réhabilitation Améliorée après Chirurgie (GRACE; Francophone Group for Enhanced Recovery after Surgery), Beaumont, France (GT, PYH), Anesthesia and Perioperative Neuroscience Laboratory, GIGA-Consciousness, GIGA-Neuroscience (FB, VB), Department of Abdominal Surgery and Transplantation, Liège University Hospital, Liège, Belgium (MV, OD)

## Abstract

**BACKGROUND:**

The Pringle manoeuvre, which involves intermittent clamping of the hepatic pedicle, is a common surgical strategy employed to reduce blood loss during liver resection. Although its impact on intra-operative glucose levels has been documented, its contribution to postoperative hyperglycaemia remained an untested hypothesis.

**OBJECTIVES:**

The primary aim of this study was to determine whether hepatic vascular clamping was associated with increased postoperative hyperglycaemia within 24 h after liver surgery. Secondary aims included examining the association between postoperative hyperglycaemia and infectious complications and length of hospital stay.

**DESIGN:**

A retrospective observational cohort study.

**SETTING:**

Single tertiary university hospital. The study period extended from January 2020 to June 2022.

**PATIENTS:**

The study sample comprised 163 adult patients who underwent elective liver resection. Patients were grouped according to the intra-operative use of the Pringle manoeuvre: 107 patients underwent clamping (Pringle group), while 56 patients did not undergo clamping (No-Pringle group). No patients were excluded, and data were complete for all individuals included.

**MAIN OUTCOME MEASURES:**

The primary outcome was the occurrence of postoperative hyperglycaemia, defined as blood glucose at least 10.0 mmol l^−1^ (180 mg dl^−1^) within 24 h after surgery. Secondary outcomes included postoperative infectious complications and length of hospital stay.

**RESULTS:**

Postoperative hyperglycaemia occurred in 55.1% of patients in the Pringle group compared to 23.2% in the No-Pringle group (*P* < 0.001). Hepatic clamping was independently associated with postoperative hyperglycaemia (adjusted odds ratio 2.91, 95% confidence interval 1.06 to 8.92). As secondary findings, a higher incidence of postoperative infections was observed in the Pringle group (23.4 vs. 5.4%, *P* = 0.007), and the median hospital length of stay was longer (4 [2 to 8] vs. 2 [1 to 6] days, *P* = 0.003).

**CONCLUSIONS:**

The Pringle manoeuvre was associated with increased postoperative hyperglycaemia within 24 h after liver surgery. These findings underscore the necessity for proactive intra-operative and postoperative glucose control strategies as an integral component of peri-operative management in hepatic surgery.

## Graphical abstract

**Figure:**
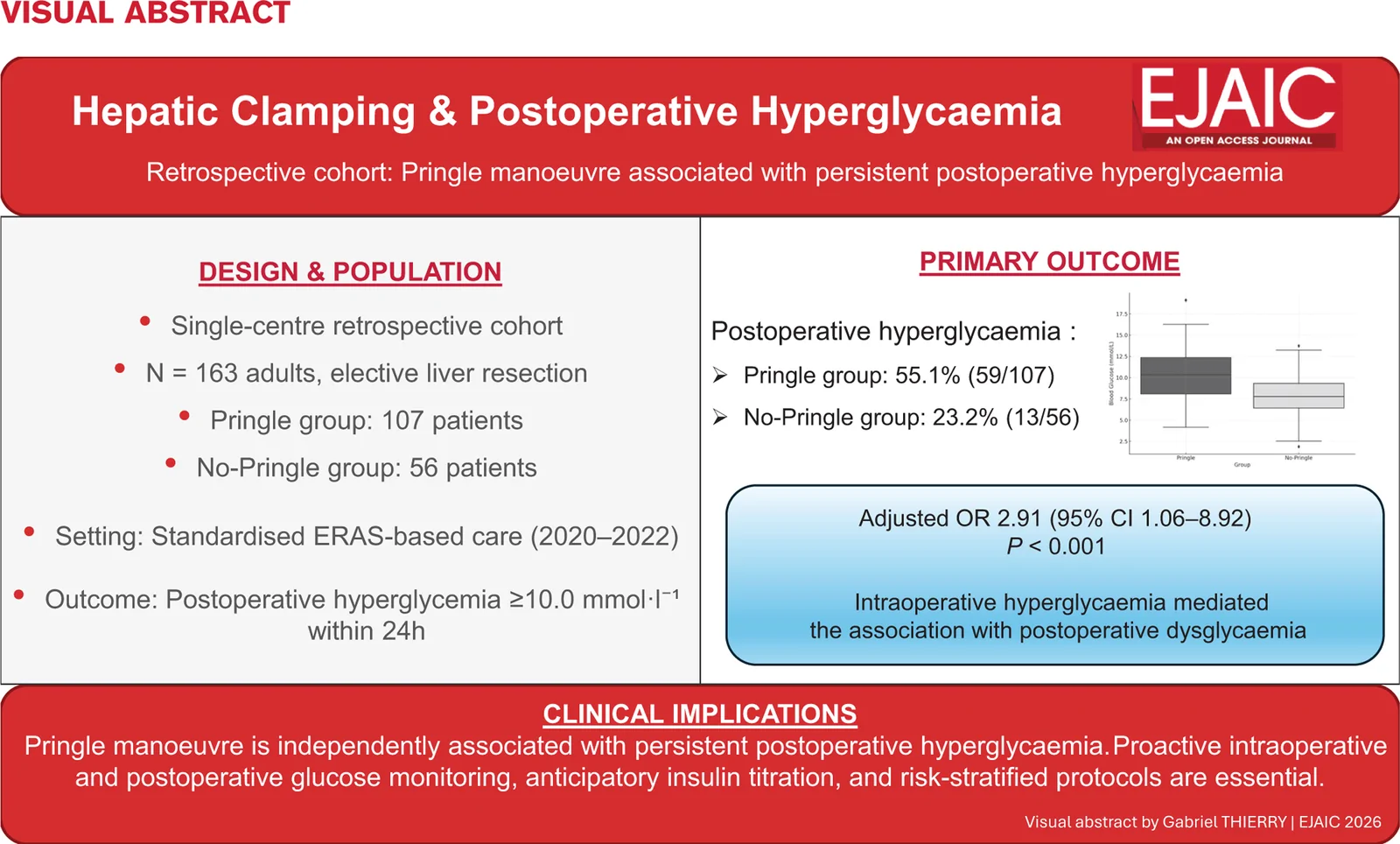

**KEY POINTS**

- Hepatic vascular clamping (Pringle manoeuvre) during liver resection is associated with early postoperative hyperglycaemia (within 24 h), occurring in 55.1% of clamped patients vs. 23.2% of nonclamped patients.
- Multivariate logistic regression analysis confirmed hepatic clamping and diabetes mellitus as independent predictors of postoperative hyperglycaemia.
- Mediation analysis demonstrated that intra-operative hyperglycaemia is a key mediator linking hepatic clamping to postoperative hyperglycaemic disturbances.
- Although secondary analyses identified associations between postoperative hyperglycaemia and infectious complications, prospective studies are needed to clarify causality in this relationship.
- These findings support the implementation of proactive intra-operative and postoperative glucose control strategies as an integral component of peri-operative management in hepatic surgery.

## Introduction

Liver surgery is one of the most haemorrhagic abdominal procedures, requiring advanced techniques to minimise intra-operative bleeding.^1^ Despite improvements in surgical techniques such as laparoscopic approaches or ultrasonic aspirators, significant bleeding (>500 ml) still occurs in this type of surgery.^2^ In this context, the Pringle manoeuvre, first described by Dr Pringle in 1906, remains a cornerstone of haemostasis in liver surgery.^3^^,^^4^

The Pringle manoeuvre involves intermittent clamping of the hepatic pedicle that contains the hepatic artery, the portal vein and the common bile duct. This technique temporarily interrupts hepatic perfusion and effectively reduces intra-operative bleeding, at the cost of liver ischaemia.

Intermittent clamping manoeuvres induce significant metabolic disturbances, particularly regarding glucose homeostasis. Both animal and human studies have shown that pedicle release is associated with hyperglycaemic peaks.^5^^,^^6^ The specific biochemical mechanisms underlying this phenomenon remain incompletely understood but are likely to involve a massive glucose release from the hepatocytes through glycogenolysis and/or neo-glycogenolysis.^7^ Repeated Pringle manoeuvres can lead to severe hyperglycaemia, reinforcing the need to anticipate and proactively control these metabolic challenges, and often requiring aggressive intravenous insulin therapy with rapidly acting agents.

Although intra-operative hyperglycaemia during liver surgery with Pringle manoeuvres is well documented,^6^^,^^8^ no previous studies have specifically investigated the impact of the Pringle manoeuvre on postoperative glycaemic control and its potential contribution to morbidity in patients undergoing liver surgery. This is a key issue, as persistent peri-operative hyperglycaemia has been identified as a major risk factor for adverse outcomes in major abdominal surgery.^9^^–^^14^ In addition, several studies have shown that postoperative hyperglycaemia has an independent effect on infectious morbidity, with odds ratios ranging from 1.5 to 2.5 in large cohorts.^11^^,^^12^^,^^15^

To fill this gap, we aimed to evaluate the relationship between hepatic vascular clamping and postoperative hyperglycaemia, hypothesising that metabolic disturbances induced by repeated Pringle manoeuvres may increase the risk of postoperative hyperglycaemia.

## Materials and methods

### Patient data

Ethical approval for this study (Ethical Committee No. 2024/342) was provided by the Hospital-Faculty Ethics Committee of the University Hospital of Liège, Belgium (Chairperson Prof. D. Ledoux) on 3 September 2024. This retrospective, single-centre cohort study included adult patients who underwent hepatic resection between January 2020 and June 2022 at the University Hospital of Liège. The ethics committee waived the requirement for written informed consent due to the retrospective nature of the study and full anonymisation of patient data prior to analysis. Patients were identified from a previous study database^16^ and were divided into two groups according to the intra-operative use (Pringle group) or omission (No-Pringle group) of the Pringle manoeuvre. At our institution, the Pringle manoeuvre is not systematically applied during liver resections. The decision to perform hepatic inflow occlusion is left to the surgeon's discretion and may be planned pre-operatively or adopted intra-operatively, depending on the anticipated complexity of the procedure and intra-operative events. This institutional practice underlies our capacity to gather data from patients who have undergone liver surgery, either with or without the Pringle manoeuvre. No patient had to be excluded, as complete data were available for all individuals. Data were retrospectively retrieved from electronic medical records using the hospital information system OmniPro (version 4.6.5.b, Xperthis/ZORGI, Belgium), which integrates laboratory, clinical and therapeutic data. All glycaemic values were extracted from arterial blood gas measurements obtained *via* point-of-care testing (i-STAT, Abbott Diagnostics, Illinois, USA). Insulin administration records were obtained from nursing documentation and pharmacy records. All data were entered into a password-protected Excel file (Microsoft Excel 2019; Microsoft Corporation, Redmond, Washington, USA) and verified for accuracy by the principal investigator. The variables included the American Society of Anesthesiologists (ASA) physical status score, sex, age, weight, height, pre-operative comorbidities, Child-Pugh and Model for End-stage Liver Disease (MELD) scores, surgical approach (laparotomy vs. laparoscopy), surgery duration, pathology, peri-operative glycaemic values and insulin treatment details. Data collection and database entry were supervised by the principal investigator and stored in an encrypted Excel file on a password-protected computer. Each patient was assigned a reference number to ensure complete anonymisation. This study was conducted and reported according to the STROBE checklist.^17^

### Peri-operative management

All patients received standardised institutional protocol, including enhanced recovery pathways, with carbohydrate loading pre-operatively, multimodal analgesia combining regional and systemic techniques, and ERAS principles, including early mobilisation and nutritional support. This standardised approach reduces variability in peri-operative care and strengthens internal validity, though the monocentric design limits external generalisability.^16^^,^^18^^,^^19^ A detailed description of our institutional liver surgery protocol is provided in Supplementary Material (Appendix 5). This protocol, which is available to all involved clinicians, includes enhanced recovery pathways including a carbohydrate load, 400 ml of 12.5% glucose, for example apple juice, 2 h before anaesthesia induction (except for patients with insulin-requiring diabetes mellitus or known gastroparesis), pre-operative nutritional therapy if needed, peri-operative antibiotic prophylaxis following standard guidelines, prevention of hypothermia, laparoscopic approach whenever feasible. General anaesthesia was performed using sevoflurane or propofol and neuromuscular blockade using rocuronium. Multimodal analgesia, combining bilateral subcostal transversus abdominis plane (TAP) block and systemic agents, including paracetamol, tramadol, intravenous lidocaine, magnesium, NSAIDs, ketamine and clonidine when indicated. Epidural analgesia was not used, even in laparotomy cases. In some cases, intrathecal morphine was used when coagulation permitted. In our institution, all patients in this cohort received a standardised single intravenous dose of 8 mg dexamethasone at induction of anaesthesia, administered before surgical incision, except in cases of uncontrolled insulin-requiring diabetes. Peri-operative glycaemic monitoring was performed by arterial blood gas analysis, using an arterial catheter placed routinely in view of the bleeding risk. Targeted blood glucose measurements were conducted a few minutes after each declamping to promptly detect and manage glycaemic spikes. Postoperative measurements were obtained within the first 24 h after surgery, with timing determined by standard clinical protocols in the postanesthesia care unit (PACU) and on the surgical ward. In routine practice, postoperative glucose is usually checked on arrival in the PACU (0 h), and again approximately 6 h and between 12 and 24 h after surgery, with additional measurements if clinically indicated. Postoperative hyperglycaemia was defined as blood glucose at least 10.0 mmol l^−1^ (180 mg dl^−1^) occurring within this first 24-h window, and for the primary analysis, the peak glucose value recorded during this period was used as the postoperative glycaemic endpoint. The therapeutic goal was to maintain intra-operative and postoperative blood glucose levels at or below 10 mmol l^−1^ (≤180 mg dl^−1^). Insulin doses administered to treat intra-operative and postoperative hyperglycaemia followed institutional protocols based on the most recent international recommendations.^20^^,^^21^ Hypoglycaemia (defined as blood glucose less than 3.9 mmol l^−1^ (or 70 mg dl^−1^) was treated with an intravenous administration of a 30% glucose solution.

### Endpoints

The primary endpoint was to evaluate the impact of the Pringle manoeuvres on postoperative hyperglycaemia rates within the first 24 h after liver surgery. Hyperglycaemia was defined as blood glucose at least 10 mmol l^−1^ (≥180 mg dl^−1^), while severe hyperglycaemia was defined as blood glucose at least 13.9 mmol l^−1^ (≥250 mg dl^−1^).^14^^,^^22^

Secondary endpoints included the potential impact of Pringle manoeuvre-induced hyperglycaemia on postoperative complications, particularly infectious complications and length of hospital stay. Postoperative complications were classified according to the European Perioperative Clinical Outcome (EPCO) definitions.^23^ Infectious complications included pneumopathy, urinary tract infection, abdominal wall infection, deep intra-abdominal abscess, peritonitis, angiocholitis, sepsis and *Clostridium difficile* infection. Surgical biliary complications (such as bilioma and biliary fistula) and intestinal fistulae were classified as surgical complications and analysed separately from infectious events, although they could be secondarily complicated by infection. Liver-related complications included acute liver failure and clinically significant ascites, and other organ complications included acute kidney injury (AKI). All complications were graded according to the Clavien-Dindo classification system, and the composite outcome ‘overall postoperative morbidity’ included all complications of Clavien–Dindo Grade I or higher. Additional outcomes included unscheduled hospital readmission rates and death, 30 and 90 days after surgery.

### Statistical analysis

Given the pre-existing database of 163 patients, a statistical power calculation was performed using G*Power (version 3.1.9.2, Franz Faul, Universität Kiel, Germany) to ensure that the sample size was sufficient to detect a significant difference in the incidence of postoperative hyperglycaemia between the Pringle and No-Pringle group. On the basis of preliminary data from our institution, postoperative hyperglycaemia was expected to occur in about 55% of patients with a Pringle manoeuvre vs. 23% of patients without clamping (absolute difference ≈32 percentage points). A post-hoc power calculation using these estimates, an alpha level of 0.05 and our actual sample size of 163 patients (107 Pringle, 56 No-Pringle) indicated that the study had 98.3% power to detect this difference in the primary outcome.

Descriptive analyses were performed for all collected variables, stratified by groups. Normality of distribution for quantitative variables was assessed numerically (mean vs. median comparison), graphically (histograms and quantile-quantile plots) and using the Shapiro–Wilk test. Data are presented as mean (standard deviation) or median (interquartile range) and analysed using unpaired two-tailed Student's *t*-test or Mann–Whitney *U* test for parametric and nonparametric variables, respectively. Categorical variables were analysed using Fisher's Exact Test and reported as percentages (%).

Univariate logistic regressions were first performed for each clinically relevant variable. All variables with potential clinical impact or statistical relevance were then included in a multivariable logistic regression model. To minimise overfitting and preserve interpretability, no automatic stepwise selection was used, given its propensity to generate unstable models and biased estimates.^24^ Covariates were therefore preselected based *a priori* on clinical plausibility and prior evidence from the literature, including age, sex,^12^^,^^15^ ASA physical status,^11^ diabetes,^25^ hepatic steatosis,^26^ intra-operative corticosteroid use,^27^^,^^28^ surgical approach (laparotomy)^29^ and hepatic vascular clamping.^30^ Operative duration at least 180 min was examined descriptively as a marker of surgical complexity but was not retained in the multivariable model for postoperative hyperglycaemia, as its independent association with this outcome is not consistently supported in the literature ^11^^,^^12^^,^^25^^,^^26^^,^^28^ and was not significant in our dataset, despite a strong relationship with intra-operative hyperglycaemia, in line with the physiological stress response to major surgery.^31^^–^^33^ Also, a subgroup analysis was performed among nondiabetic patients undergoing laparoscopic surgery to exclude diabetes mellitus and laparotomy from the model and further try to evaluate the specific effect of hepatic clamping on the occurrence of postoperative hyperglycaemia.

Analyses were performed using R version 4.3.1 (R Foundation for Statistical Computing, Vienna, Austria).

## Results

**Fig. 1:**
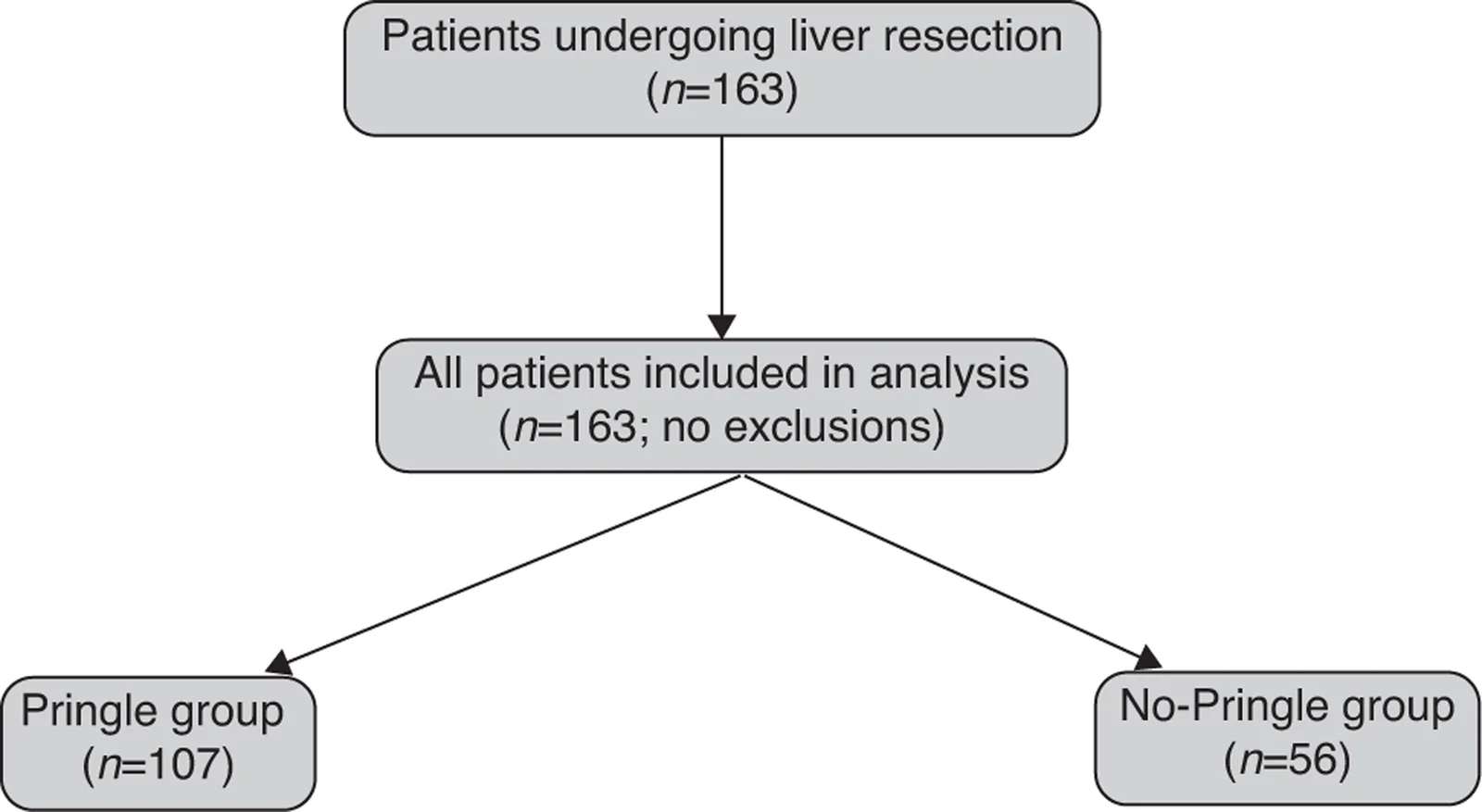
Flow diagram of patient selection and allocation.

This study included 163 adult patients who underwent elective hepatic resection at our institution between January 2020 and June 2022. A flow diagram detailing patient selection and group allocation is presented in Fig. 1. All 163 eligible patients were included without exclusion, and complete data were available for all individuals.

All 163 patients who underwent liver resection between January 2020 and June 2022 were included. Patients were allocated to the Pringle group (*n* = 107) or No-Pringle group (*n* = 56) based on intra-operative clamping.

### Patients and surgery characteristics

Table 1 shows pre-operative and intra-operative data. Operative characteristics differed between groups. Open surgical approaches were significantly more frequent in the Pringle group than in the No-Pringle group (69.2 vs. 30.4%, respectively, *P* < 0.001, Fisher's Exact test). The rate of major liver resections was higher in the Pringle group compared to the No-Pringle group (47.7 vs. 19.6%, *P* < 0.001, Fischer's Exact test). Operative duration was significantly longer in the Pringle group, with a median [IQR] of 166 [129.5 to 207] min, compared to 105 [58 to 184.25] min in the No-Pringle group (*P* < 0.001, Mann–Whitney *U*-test).

**Table 1:** Pre-operative and intra-operative characteristics according to Pringle manoeuvre use

	Overall (*n* = 163)	Pringle (*n* = 107)	No-Pringle (*n* = 56)	*P*
ASA physical status score	2.0 [2.0 to 3.0]	2.0 [2.0 to 3.0]	2.0 [1.0 to 3.0]	0.109
1	28 (17.2)	15 (26.8)	13 (12.1)	
2	82 (50.3)	26 (46.4)	56 (52.3)	
3	52 (31.9)	15 (26.8)	37 (34.6)	
4	1 (0.6)	0 (0.0)	1 (0.9)	
Female sex	88 (63.99)	52 (48.60)	36 (64.29)	0.069
Age (years)	61.0 [52.5 to 70.0]	63.0 [53.0 to 70.0]	60.0 [50.0 to 69.0]	0.318
Weight (kg)	71.0 [61.5 to 82.0]	73.0 [62.0 to 82.5]	68.5 [60.75 to 80.0]	0.351
Height (cm)	168.0 [162.0 to 175.0]	170.0 [164.0 to 175.0]	165.0 [160.0 to 171.25]	0.094
BMI (kg m^−2^)	25.31 [22.32 to 28.075]	25.21 [22.17 to 28.21]	25.38 [22.98 to 27.79]	0.879
Obesity (BMI>30 kg m^−2^)	21 (12.88)	14 (13.08)	7 (12.50)	1
Diabetes mellitus	35 (21.47)	26 (24.30)	9 (16.07)	0.315
-Insulin-requiring diabetes	8 (4.91)	6 (5.61)	2 (3.57)	0.716
Dyslipidaemia	33 (20.25)	22 (20.56)	11 (19.64)	1
Child-Pugh score	5.0 [5.0–5.0]	5.0 [5.0–5.0]	5.0 [5.0–5.0]	0.226
MELD score	6.0 [6.0 to 8.0]	7.0 [6.0 to 8.0]	6.0 [6.0 to 7.0]	0.239
Open surgery	47 (28.83)	41 (38.32)	6 (10.71)	< 0.001
Major liver resection (3 liver segments or more)	60 (36.8)	46 (43.0)	14 (25.0)	0.037
Duration of surgery (3 categories) (min)	154 [98.0 to 203.5]	166.0 [129.5 to 207.0]	105.0 [58 to 184.25]	< 0.001
- <180 min	104 (63.80)	64 (59.81)	40 (71.43)	0.171
- ≥180 min	59 (36.20)	43 (40.19)	16 (28.57)	0.171
Healthy liver	109 (66.87)	68 (63.55)	41 (73.21)	0.226
Steatotic liver	22 (13.50)	14 (13.08)	8 (14.29)	0.814
Hepatic fibrosis	18 (11.04)	14 (13.08)	4 (7.14)	0.302
Cirrhosis	13 (7.98)	11 (10.28)	2 (3.57)	0.222
Pre-operative carbohydrates loading	105 (64.42)	69 (64.49)	36 (64.29)	1
Intra-operative corticosteroids	149 (91.41)	98 (91.59)	51 (91.07)	1
Total duration of Pringle manoeuvres (min)	40.0 [20.0 to 58.5]	40.0 [20.0 to 58.75]	0.0 [0.0 to 0.0]	–

Data are expressed as median [interquartile range] for continuous variables and number (percentage) for categorical variables. Comparisons between groups were performed using the Mann–Whitney *U* test for nonnormally distributed continuous variables, the Student's *t*-test for normally distributed continuous variables and Fisher's Exact test for categorical variables, as appropriate

ASA, American Society of Anesthesiologists.

### Peri-operative glucose levels

**Fig. 2:**
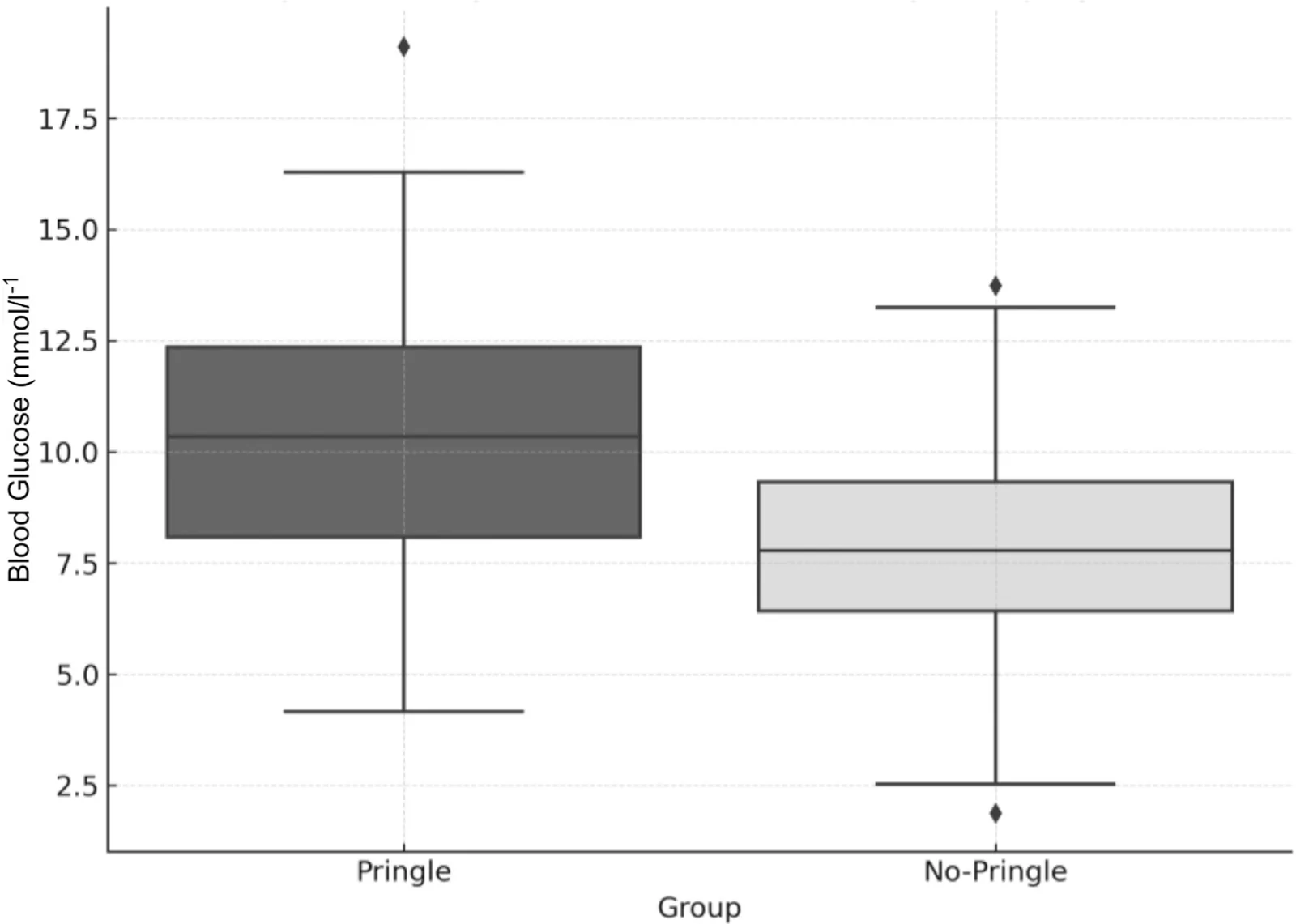
Blood glucose levels by clamping status at postoperative day 1.

Table 2 summarises peri-operative blood glucose values and therapeutic use of intravenous and subcutaneous insulin. Regarding our primary endpoint, postoperative hyperglycaemia was significantly more frequent in the Pringle group than in the No-Pringle group (55.14 vs. 23.21% respectively, *P* < 0.001, Fisher's Exact test) (Fig. 2), although there was no difference in the rate of postoperative severe hyperglycaemia between groups (5.61 vs. 5.36%, *P* = 1.000, Fisher's Exact test). Peak postoperative blood glucose levels had a median [IQR] of 10.8 [8.2 to 13.1] mmol l^−1^ (194.4 [147.6 to 235.8] mg dl^−1^) in the Pringle group and 7.5 [5.3 to 10.8] mmol l^−1^ (135 [95.4 to 194.4] mg dl^−1^) in the No-Pringle group (*P* < 0.001, Mann–Whitney *U*-test). Postoperative hyperglycaemia also necessitated more frequent insulin use in the Pringle group (47.66 vs. 19.64% in the No-Pringle group, *P* = 0.001, Fisher's Exact test), with a predominance of subcutaneous administration. Among patients with postoperative hyperglycaemia, the insulin dosage required for correction was not significantly different between groups (median [IQR]: 6.0 [4.0 to 14.5] in the Pringle group vs. 8.0 [6.0 to 16.0] in the No-Pringle group, *P* = 0.252, Fisher's Exact test). The incidence of postoperative hypoglycaemia was similar between groups (2.80% in the Pringle group vs. 1.79% in the No-Pringle group, *P* = 1.000, Fisher's Exact test).

**Table 2:** Glycaemic parameters and insulin use according to Pringle manoeuvre use

	Overall (*n* = 163)	Pringle (*n* = 107)	No-Pringle (*n* = 56)	*P*
Intra-operative peak glycaemia (mmol l^−1^)	13.8 [11.6 to 17.1]	15.4 [12.3 to 17.5]	11.4 [10.2 to 13.8]	**0.001** (Student's *t-*test)
Intra-operative hyperglycaemia (> 10 mmol l^−1^) (*n*)	103 (63.19)	84 (78.50)	19 (33.93)	**<0.001** (Fisher's Exact test)
Intra-operative severe hyperglycaemia (> 13.9 mmol l^−1^) (*n*)	33 (20.25)	31 (28.97)	2 (3.57)	**<0.001** (Fisher's Exact test)
Intra-operative insulin use	73 (44.79)	64 (59.81)	9 (16.07)	**<0.001** (Fisher's Exact test)
-Intravenous insulin	72 (44.17)	64 (59.81)	8 (14.29)	**<0.001** (Fisher's Exact test)
-Subcutaneous insulin	2 (1.23)	1 (0.93)	1 (1.79)	1 (Fisher's Exact test)
Intra-operative insulin dose (IU)	9.55 [6.0 to 13.75]	9.0 [6.0 to 14.0]	10.0 [8.3 to 10.0]	0.967 (Mann–Whitney *U* test)
Postoperative peak glycaemia (mmol l^−1^)	9.7 [7.4 to 12.3]	10.8 [8.2 to 13.1]	7.5 [5.3 to 10.8]	**<0.001** (Mann–Whitney *U* test)
Postoperative nadir glycaemia (mmol l^−1^)	6.3 [5.1 to 7.8]	6.6 [5.4 to 7.9]	5.5 [4.8 to 6.9]	**0.005** (Mann–Whitney *U* test)
Postoperative hyperglycaemia (> 10 mmol l^−1^) (*n*)	72 (44.17)	59 (55.14)	13 (23.21)	**<0.001** (Fisher's Exact test)
Postoperative severe hyperglycaemia (> 13.9 mmol l^−1^) (*n*)	9 (5.52)	6 (5.61)	3 (5.36)	1 (Fisher's Exact test)
Postoperative hypoglycaemia (< 3,9 mmol l^−1^) (*n*)	4 (2.45)	3 (2.80)	1 (1.79)	1 (Fisher's Exact test)
Postoperative insulin use	62 (38.04)	51 (47.66)	11 (19.64)	**0.001** (Fisher's Exact test)
-Intravenous insulin use	12 (7.36)	10 (9.35)	2 (3.57)	0.222 (Fisher's Exact test)
-Subcutaneous insulin use	52 (31.90)	43 (40.19)	9 (16.07)	**0.002** (Fisher's Exact test)
Postoperative insulin dose (IU)	6.0 [4.0 to 15.0]	6.0 [4.0 to 14.5]	8.0 [6.0 to 16.0]	0.252 (Mann–Whitney *U* test)

Bold values indicate statistically significant differences between groups (*P* &lt; 0.05).

Data are expressed as median [interquartile range] for continuous variables and number (percentage) for categorical variables. Comparisons between groups were performed using the Mann–Whitney *U* test for nonnormally distributed continuous variables, the Student's *t*-test for normally distributed continuous variables and Fisher's Exact test for categorical variables, as appropriate.

Boxplot showing postoperative blood glucose levels (in mmol l^-1^) in patients with and without hepatic clamping (Pringle manoeuvre). Median glucose levels were higher in the Pringle group (*P* < 0.001, Mann–Whitney *U* test). The central line represents the median, the box represents the interquartile range [IQR], and whiskers extend to 1.5 × IQR.

Intra-operatively, hyperglycaemia (78.50% in the Pringle group and 33.93% in the No-Pringle group, *P* < 0.001, Fisher's Exact test) and severe hyperglycaemia (28.97% in the Pringle group and 3.57% in the No-Pringle group, *P* < 0.001, Fisher's Exact test) were significantly more frequent in the Pringle group, with peak blood glucose levels at a median [IQR] of 15.4 mmol l^−1^ (277.2 mg dl^−1^) [12.3 to 17.5] as compared to 11.4 mmol.l^−1^ (205.2 mg dl^−1^) [10.2 to 13.8] in the No-Pringle group (*P* = 0.001, Mann–Whitney *U* test). Consequently, intra-operative insulin therapy was required significantly more often in the Pringle group (59.81 vs. 16.07% in the No-Pringle group, *P* < 0.001, Fisher's Exact test), predominantly through intravenous administration. However, among patients who developed intra-operative hyperglycaemia, the insulin dosage required for correction was similar between groups (median (IQR): 9.0 [6.0 to 14.0] IU in the Pringle group vs. 10.0 [8.3 to 10.0] IU in the No-Pringle group, *P* = 0.432, Mann–Whitney *U*-test).

Also, when stratifying patients according to operative duration (<3 vs. ≥3 h), intra-operative hyperglycaemia occurred significantly more frequently in patients undergoing longer procedures. Specifically, intra-operative hyperglycaemia was observed in 79.7% (47/59) of patients with surgeries lasting at least 3 h, compared to 53.8% (56/104) in those with shorter procedures (*P* = 0.001, Fisher's Exact test). In contrast, postoperative hyperglycaemia occurred in 50.8% (30/59) of patients in the at least 3 h group vs. 40.4% (42/104) in the less than 3 h group, a difference that was not statistically significant (*P* = 0.251, Fisher's Exact test).

### Univariate and multivariate logistic regressions

**Fig. 3:**
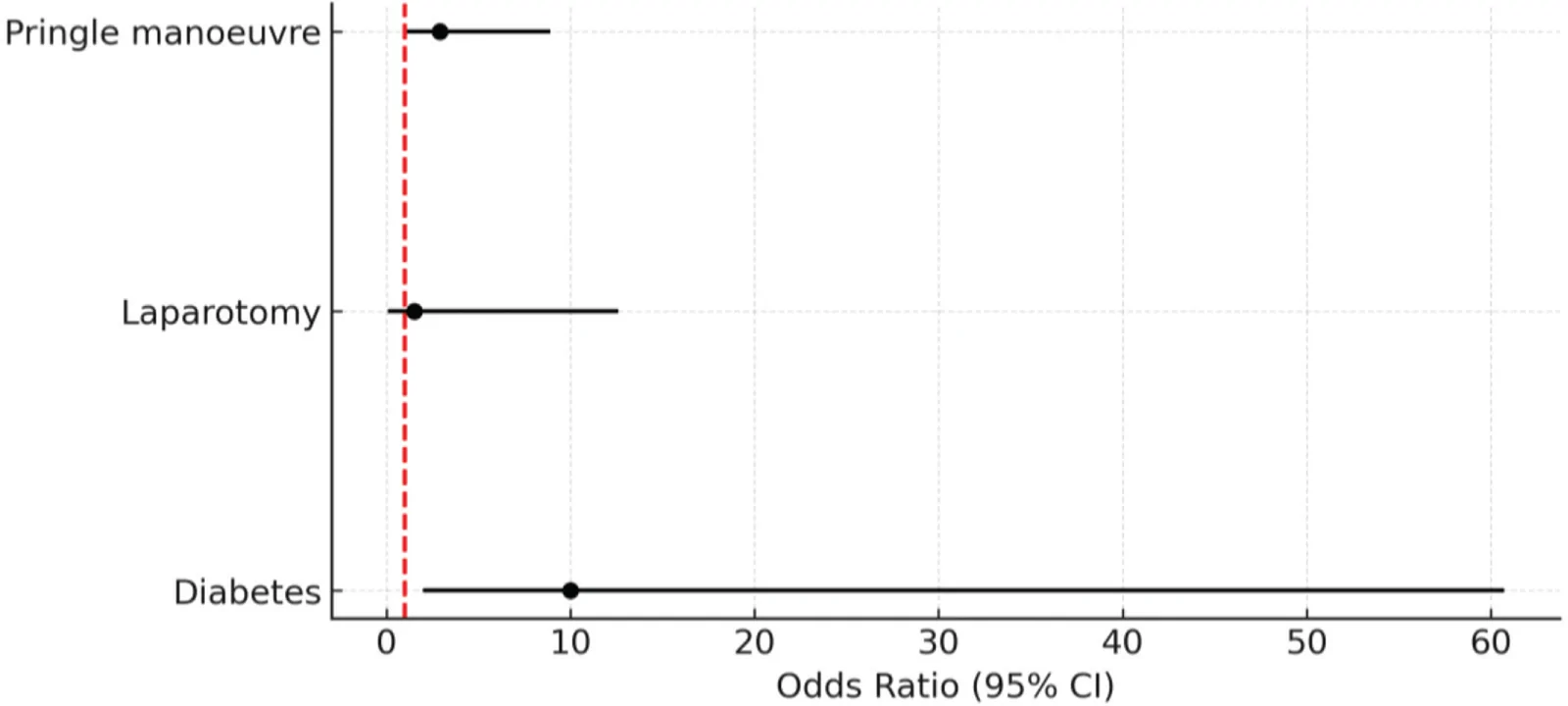
Multivariate logistic regression – predictors of postoperative hyperglycaemia.

Univariate logistic regressions (Appendix 1) showed that a past medical history of diabetes, OR = 7.64 (95% CI, 3.24 to 20.28); laparotomy surgery, OR = 3.1 (95% CI, 1.55 to 6.40), and the Pringle manoeuvre, OR = 4.07 (95% CI, 2.01 to 8.68), were significantly associated with the occurrence of postoperative hyperglycaemia. The final multivariate model (Fig. 3 and Appendix 2) confirmed that hepatic clamping, OR = 2.91 (95% CI, 1.06 to 8.92) and diabetes, OR = 10.00 (95% CI, 1.97 to 60.73), were independent predictors of postoperative hyperglycaemia, while laparotomy was not, OR = 1.50 (95% CI, 0.07 to 12.60).

Forest plot displaying odds ratios (ORs) with 95% confidence intervals (95% CIs) from the final multivariate logistic regression model. Diabetes OR = 10.00 (1.97 to 60.73); *P* = 0.007, and the use of the Pringle manoeuvre OR = 2.91 (1.06 to 8.92); *P* = 0.046, were significantly associated with postoperative hyperglycaemia, while laparotomy was not OR = 1.50 (0.07 to 12.60); *P* = 0.736.

In the nondiabetic, laparoscopic subgroup (*n* = 91, appendix 3), hepatic clamping remained associated with postoperative hyperglycaemia, OR = 3.09 (95% CI, 1.02 to 9.41).

A mediation analysis (Appendix 4) confirmed that intra-operative hyperglycaemia mediated the association between clamping and postoperative hyperglycaemia as the direct effect of clamping was weak after adjustment, OR = 1.77 (95% CI, 0.74 to 4.22), whereas intra-operative hyperglycaemia was strongly predictive, OR = 19.99 (95% CI, 4.91 to 34.45).

### Morbidity and mortality

Table 3 summarises postoperative outcomes. The incidence of overall postoperative morbidity was significantly higher in the Pringle group than in the No-Pringle group (44.86 vs. 26.79%, respectively, *P* = 0.028, Fisher's Exact test). Infectious complications were more frequent in the Pringle group (23.4 vs. 5.4% in the No-Pringle group, *P* = 0.004, Fisher's Exact test). The length of hospital stay was also longer in the Pringle group (median [IQR]: 4 days [2 to 8] vs. 2 days [1 to 6] in the No-Pringle group, *P* = 0.003, Mann–Whitney *U*-test). The majority of patients were admitted postoperatively to regular surgical wards rather than to intermediate care or ICUs (78.9% overall; 85/107 in the Pringle group and 41/56 in the No-Pringle group, *P* = 0.39; Table 3). Finally, there were no differences between groups concerning unplanned readmissions and death at 30 and 90 days.

**Table 3:** Postoperative outcomes and complications according to Pringle manoeuvre use

	Overall (*n* = 163)	Pringle (*n* = 107)	No-Pringle (*n* = 56)	*P*
Any postoperative complications	63 (38.65)	48 (44.86)	15 (26.79)	**0.028**
Infectious complications	28 (17.18)	25 (23.36)	3 (5.36)	**0.004**
- Angiocholitis	0.0 [0.0 to 0.0]	0.0 [0.0 to 0.0]	0.0 [0.0 to 0.0]	–
-*Clostridium difficile* infection	1 (0.61)	1 (0.93)	0 (0.00)	1
-Deep abscess	18 (11.04)	15 (14.02)	3 (5.36)	0.118
-Abdominal wall infection	4 (2.45)	3 (2.80)	1 (1.79)	1
-Peritonitis	3 (1.84)	1 (0.93)	2 (3.57)	0.272
-Pneumopathy	4 (2.45)	4 (3.74)	0 (0.00)	0.300
-Urinary tract infection	2 (1.23)	2 (1.87)	0 (0.00)	0.546
-Sepsis	5 (3.07)	3 (2.80)	2 (3.57)	1
Other complications				
-Bilioma	21 (12.88)	17 (15.89)	4 (7.14)	0.143
-Biliary fistula	13 (7.98)	11 (10.28)	2 (3.57)	0.222
-Liver failure	10 (6.13)	7 (6.54)	3 (5.36)	1
-Ascites	6 (3.68)	4 (3.74)	2 (3.57)	1
-Deep haematoma	7 (4.29)	4 (3.74)	3 (5.36)	0.692
-Acute kidney injury	6 (3.68)	5 (4.67)	1 (1.79)	0.665
-Intestinal fistula	1 (0.61)	1 (0.93)	0 (0.00)	1
Complication severity (Clavien Dindo classification)				0.457
-Grade I	12 (7.36)	9 (8.41)	3 (5.36)	
-Grade II	32 (19.63)	25 (23.36)	7 (12.5)	
-Grade IIIa	6 (3.68)	5 (4.67)	1 (1.79)	
-Grade IIIb	11 (6.51)	7 (6.54)	4 (7.14)	
-Grade IVa	1 (0.61)	1 (0.93)	0 (0.00)	
-Grade IVb	0 (0.00)	0 (0.00)	0 (0.00)	
-Grade V (death)	1 (0.61)	1 (0.93)	0 (0.00)	
Unscheduled early re-operation	7 (4.29)	5 (4.67)	2 (3.57)	1
Postoperative stay in intermediate care	31 (19.02)	24 (22.43)	7 (12.50)	0.145
Postoperative stay in intensive care	3 (1.84)	3 (2.80)	0 (0.00)	0.552
Length of hospital stay (days)	4.0 [1.0 to 7.0]	4.0 [2.0 to 8.0]	2.0 [1.0 to 6.0]	**0.003**
Unplanned hospital readmission within 30 days	24 (14.72)	19 (17.76)	5 (8.93)	0.165
Unplanned hospital readmission within 90 days	30 (18.40)	23 (21.50)	7 (12.50)	0.203
Delay to readmission (days)	14.0 [9.0 to 30.0]	15.0 [9.0 to 30.0]	12.0 [9.0 to 33.5]	0.698
Death within 30 days	1 (0.61)	1 (0.93)	0 (0.00)	1
Death within 90 days	3 (1.84)	3 (2.80)	0 (0.00)	0.552

Bold values indicate statistically significant differences between groups (*P* &lt; 0.05).

Data are expressed as median [interquartile range] for continuous variables and number (percentage) for categorical variables. Comparisons were performed using the Mann–Whitney *U* test for continuous variables and Fisher's Exact test for categorical variables.

## Discussion

This study confirms that hepatic vascular clamping (Pringle manoeuvre) is significantly associated with intra-operative hyperglycaemia, including severe hyperglycaemia, as previously described in both animal and human studies.^5^^,^^34^ Our multivariate model demonstrates an independent effect of clamping (OR 2.91) and diabetes mellitus (OR 10), while laparotomy may not exert an independent influence in this respect. Although hepatic vascular clamping often remains necessary to ensure adequate haemostasis in complex liver resections, the associated hyperglycaemic response should be regarded as a predictable and potentially manageable metabolic consequence rather than an unavoidable complication.

The strength of our study lies in our primary outcome findings, wherein we established a clear and statistically significant relationship between Pringle manoeuvres and postoperative hyperglycaemia. Despite the modest sample size, our study benefits from complete standardisation of peri-operative management through ERAS protocols and multimodal analgesia. All patients received consistent enhanced recovery pathways, carbohydrate loading, regional anaesthesia techniques and systematic peri-operative monitoring. This standardised institutional approach reduces variability and confounding elements from varying peri-operative care, thereby strengthening the internal validity of our findings regarding the specific effect of hepatic clamping on postoperative hyperglycaemia. Although prior research has reported intra-operative hyperglycaemic spikes linked to hepatic ischaemia–reperfusion, our study seems to show that these disturbances persist postoperatively. Our mediation analysis ^35^ suggests that intra-operative hyperglycaemia may act as a key factor influencing the relationship between hepatic clamping and postoperative glycaemic disturbances. These findings emphasise the importance of structured intra-operative and postoperative glucose control strategies to mitigate clamp-related dysglycaemia and minimise the metabolic impact of hepatic clamping. Indeed, current peri-operative glycaemic management strategies primarily focus on correcting postoperative hyperglycaemia rather than already addressing it intra-operatively. The development of more proactive intra-operative glucose management protocols, adapted to liver surgery with Pringle manoeuvres, could prove useful. Emerging technologies, such as real-time continuous glucose monitoring and closed-loop insulin delivery systems, could provide valuable tools for optimising peri-operative glycaemic control in this setting.^36^ A noteworthy observation in our cohort is that insulin doses did not differ significantly between groups, despite a markedly higher incidence of both intra-operative and postoperative hyperglycaemia in patients undergoing hepatic clamping. This pattern suggests that current insulin titration practices may not fully anticipate the magnitude or persistence of the glycaemic response associated with Pringle manoeuvres, and could reflect a combination of protocol limitations, monitoring intensity and clinical inertia in ward-based care. From a pragmatic standpoint, these findings support the need to refine peri-operative glucose management algorithms for patients at risk of clamp-related dysglycaemia, including clearer anticipatory dosing strategies and more frequent postoperative measurements in high-risk individuals. Of note, in our institution, most patients were managed postoperatively on regular surgical wards despite the high-risk nature of liver surgery. This setting is associated with less intensive monitoring than intermediate or ICUs and may have contributed to delayed detection or undertreatment of hyperglycaemic episodes, particularly in patients undergoing hepatic clamping. These observations further support the need to adapt postoperative glucose monitoring intensity and insulin titration protocols to the specific metabolic risk profile of liver resection patients, rather than relying solely on standard ward-based practices. As expected, operative times were longer in the Pringle group, consistent with the greater surgical complexity of open and major hepatectomies requiring Pringle manoeuvres. The distribution analysis confirmed that hepatic clamping was more frequently applied in procedures lasting at least 180 min, whereas shorter resections (such as single wedge resections) required clamping less often, with no significant difference for intermediate durations, supporting the idea that Pringle manoeuvres are mainly associated with more extensive and technically demanding procedures. Although a strong association was found between prolonged surgery and intra-operative hyperglycaemia, operative time was not independently predictive of postoperative hyperglycaemia in our data. This aligns with current literature, which, while suggesting potential effects of longer procedures on insulin delivery or glycaemic variability, does not support a direct causal relationship with postoperative dysglycaemia.^11^^,^^12^^,^^25^^,^^26^^,^^28^ Only one monocentric study reported a weak association, with an odds ratio of 1.011 per minute using a low threshold of more than 6.7 mmol l^−1^ (>120 mg dl^−1^) to define postoperative hyperglycaemia.^37^ Accordingly, operative duration was excluded from our multivariable model to maintain parsimony and robustness (see Materials and methods).

In our cohort, higher rates of infectious complications and longer hospital stay were observed in the Pringle group, in line with literature linking prolonged peri-operative hyperglycaemia to adverse postoperative outcomes.^8^^,^^11^^–^^15^^,^^25^^,^^38^^,^^39^ However, these secondary findings arose from a relatively low event rate in a retrospective design and should be interpreted with caution, serving mainly to reinforce the clinical importance of preventing and treating sustained peri-operative hyperglycaemia in this high-risk population. Prolonged exposure to elevated blood glucose levels may impair immune function, delay wound healing and exacerbate inflammatory responses, suggesting that interventions aimed at controlling intra-operative and postoperative hyperglycaemia could have downstream benefits in reducing postoperative complications, although our study was not designed to formally test these causal pathways. Although our multivariable analysis adjusted for intra-operative corticosteroid use, other peri-operative factors, including vasopressor administration, transfusion requirements and intra-operative blood loss, are also likely to influence postoperative glycaemic control and may have contributed to the observed associations. These variables should be prospectively collected and incorporated as key confounders in future studies to better disentangle the specific impact of hepatic clamping on postoperative dysglycaemia. Prospective studies with comprehensive peri-operative data are therefore required to address the point. A subgroup analysis, excluding diabetic and laparotomy patients (variables that showed a significant prediction ability with regard to the occurrence of postoperative hyperglycaemia), was performed to minimise confounding factors that could obscure the true impact of hepatic clamping on glycaemic outcomes (Appendix 3). Although these refinements allowed a more specific assessment of the metabolic consequences of Pringle manoeuvres, they also led to a reduced sample size, thereby limiting the statistical power of certain analyses.

From a clinical perspective, our findings challenge conventional assumptions regarding hepatic ischemia and metabolic responses in liver surgery. The traditional focus on hypoglycaemia as a marker of liver dysfunction ^40^ should not be applied to liver surgery using Pringle manoeuvres, as our results show a paradoxical, yet clinically significant hyperglycaemia response that appears to be associated with hepatic vascular clamping. Anaesthesiologists and surgical teams should integrate this knowledge into their peri-operative management strategies. Beyond intra-operative and postoperative considerations, there might be a role for pre-operative patient optimisation in mitigating glycaemic disturbances. Nutritional strategies, including carbohydrate loading, metabolic prehabilitation and insulin sensitivity optimisation, may help modulate peri-operative glucose metabolism.^41^

Our study has limitations. Firstly, despite the high statistical power related to our primary outcome (98.3%), it must be acknowledged that a high power does not compensate for the absence of randomisation. As this is a retrospective, observational study, potential selection biases and confounding factors cannot be entirely excluded. Secondly, our retrospective and monocentric design limits external validity. Thirdly, postoperative glycaemic assessment was limited to intermittent measurements within the first 24 h after surgery, without continuous monitoring beyond this period. More granular data on glycaemic trajectories, for example using continuous glucose monitoring over several postoperative days, would provide a richer understanding of dysglycaemia patterns and should be considered in future prospective studies. Fourthly, the relatively low incidence of complications in our cohort restricts the power of secondary analyses. Larger, multicentric prospective studies are needed to validate these findings and to assess whether specific patient subgroups, such as those with preexisting insulin resistance or metabolic syndrome, may be particularly susceptible to the deleterious effects of prolonged peri-operative hyperglycaemia. In summary, our results support a paradigm shift in the metabolic interpretation of hepatic clamping, demonstrating that the Pringle manoeuvre is associated with persistent postoperative hyperglycaemia. Although item 19 of the ERAS liver surgery protocol already acknowledges the importance of peri-operative glucose management, our findings suggest that additional attention to monitoring frequency, insulin dosing anticipation and risk stratification may be warranted specifically for patients undergoing hepatic clamping. Enhanced implementation and personalisation of existing glucose management recommendations, particularly in the postoperative period, may help to mitigate the hyperglycaemic response observed in our cohort.

## Supplementary Material

Supplemental Digital Content
