# Supplementary material for: Hepatic clamping and postoperative hyperglycaemia: A retrospective cohort study in liver surgery
Source: Eur J Anaesthesiol Intensive Care. 2026 Jun 24;5(4):e0123. doi: 10.1097/EA9.0000000000000123 (PMC13568249; doi:10.1097/EA9.0000000000000123)
Supplement: Supplementary file 1 [file ejaic-5-e0123-s001.docx]

**Appendix 1 – Univariate Logistic Regression for Postoperative Hyperglycemia**

The following univariate logistic regression models were conducted to assess potential predictors of postoperative hyperglycemia. Results are expressed as Odds Ratios (OR), 95% Confidence Intervals (CI), and p-values.

- ASA Score
  - ASA 2 vs 3 (ref): OR = 1.73; 95% CI: [0.27–1.11]; p = 0.094
  - ASA 1 vs 3 (ref): OR = 1.74; 95% CI: [0.21–1.40]; p = 0.216
- Age : OR = 1.01; 95% CI: [0.99–1.04]; p = 0.356
- Sex (female): OR = 0.68; 95% CI: [0.36–1.26]; p = 0.221
- Diabetes: OR = 7.64; 95% CI: [3.24–20.28]; p < 0.001
- Hepatic steatosis: OR = 0.86; 95% CI: [0.33–2.12]; p = 0.741
- Corticosteroid use: OR = 0.41; 95% CI: [0.12–1.24]; p = 0.122
- Laparotomy: OR = 3.11; 95% CI: [1.55–6.40]; p = 0.002
- Pringle maneuver: OR = 4.07; 95% CI: [2.01–8.68]; p < 0.001

**Appendix 2 – Multivariate Logistic Regression with Interaction Terms**

A final multivariate logistic regression model was built including two-way and three-way interaction terms between diabetes, laparotomy, and hepatic clamping (Pringle maneuver). The results are as follows:

- Intercept: OR = 0.17; 95% CI: [0.06–0.37]; p < 0.001
- Diabetes: OR = 10.00; 95% CI: [1.97–60.73]; p = 0.007
- Laparotomy: OR = 1.50; 95% CI: [0.07–12.60]; p = 0.736
- Pringle maneuver: OR = 2.91; 95% CI: [1.06–8.92]; p = 0.046
- Interaction – Diabetes × Laparotomy : OR = 847,272; 95% CI: [NA–NA] ; p = 0.988
- Interaction – Diabetes × Pringle: OR = 1.55; 95% CI: [0.16–17.99]; p = 0.711
- Interaction – Laparotomy × Pringle: OR = 2.63; 95% CI: [0.26–62.47]; p = 0.456
- Interaction – Diabetes × Laparotomy × Pringle: OR = 0.00; 95% CI: [NA–2.04×10⁷⁰]; p = 0.986

**Appendix 3 – Subgroup analysis excluding diabetic patients and laparotomy**

A subgroup analysis, excluding diabetic and laparotomy patients, assessed the impact of hepatic clamping on postoperative hyperglycemia. Among the remaining 91 patients, multivariate logistic regression confirmed that hepatic clamping significantly increased the risk of postoperative hyperglycemia (OR = 3.09; 95% CI: [1.02–9.41], p = 0.047). Other variables, including ASA score, age, hepatic steatosis, perioperative corticosteroid use, and male sex, were not significantly associated with postoperative hyperglycemia in this subgroup. The model’s Pseudo R² was 0.0689, indicating that 6.9% of the variance was explained. The Log-Likelihood Ratio Test showed that the overall model did not reach statistical significance (p = 0.3264), although hepatic clamping remained a significant variable.

**Appendix 4 – Mediation Analysis**

Mediation pathway:
Clamping → Intraoperative hyperglycemia → Postoperative hyperglycemia

**Step 1 – Total effect (path c)**

Model: Total effect of hepatic clamping on postoperative hyperglycemia

- Coefficient: 1.4026
- Odds Ratio (OR): 4.07 (95% CI: [2.01 – 8.68])
- p < 0.001

**Step 2 – Effect of clamping on intraoperative hyperglycemia (path a)**

Model: Association between hepatic clamping and intraoperative hyperglycemia

- Coefficient: 1.9618
- Odds Ratio (OR): 7.12 (95% CI: [3.46 – 14.63])
- p < 0.001

**Step 3 – Direct and indirect effects (paths c′ and b)**

Model: Association of both hepatic clamping and intraoperative hyperglycemia with postoperative hyperglycemia

Clamping (direct effect, c′):

- Coefficient: 0.5728
- Odds Ratio (OR): 1.77 (95% CI: [0.74 – 4.22])
- p = 0.195

Intraoperative hyperglycemia (indirect effect, path b):

- Coefficient: 2.5657
- Odds Ratio (OR): 12.99 (95% CI: [4.91 – 34.45])
- p < 0.001

**Appendix 5 – Institutional protocol for liver surgery

Enhanced Recovery After Hepatic Surgery (ERAS)
Dr Thierry – Kaba – Hardy – Detry**

**1. Preoperative period**

Preoperative visit
Whenever possible, the pre‑anesthesia visit should be scheduled after the surgical consultation. During this visit, the anesthesiologist reinforces the instructions and reviews the enhanced recovery protocol with the patient.

Patient information
The patient receives oral and written information from both the surgeon and the anesthesiologist about the planned hospital stay and the modalities of the enhanced recovery protocol. The importance of the patient’s active participation is emphasized. The “ERAS information” leaflet is provided during the pre‑anesthesia consultation. For patients undergoing a segmentectomy (1 to 3 segments) on a non‑cirrhotic liver, the possibility of same‑day discharge is mentioned.

Preoperative physiotherapy
A consultation with a physiotherapist is scheduled before or after the pre‑anesthesia visit. Advice and exercises for pre‑ and postoperative mobilization are explained, as well as the importance of early postoperative mobilization. For hepatectomies planned via laparotomy, preoperative respiratory physiotherapy is organized and the patient receives an incentive spirometry device.

Cessation of alcohol and tobacco use
Smoking and alcohol cessation is strongly recommended by international societies. To convince the patient, clinicians are encouraged to stress the association between smoking and suture dehiscence or incisional hernia.

Nutritional management
Some patients scheduled for liver surgery present nutritional risk factors (weight loss ≥10% over the last 6 months, BMI <18.5 kg·m⁻², serum albumin <30 g·L⁻¹). In such patients, oral nutritional supplements (e.g. Impact®, Fresubin®) are prescribed during the week preceding surgery, with dietitian input when needed.

Premedication
Routine anxiolytic premedication is avoided because it may delay postoperative recovery. Exceptions can be made on a case‑by‑case basis.

Preoperative fasting
To avoid case cancellations due to schedule changes, patients remain fasting for solids from midnight. Clear fluids (water, tea, coffee, soft drinks, pulp‑free juice) are allowed up to 2 hours before anesthesia.

Preoperative blood tests
Recent laboratory tests (less than 1 month old) must be available, including full blood count, coagulation profile, liver function tests, and initial blood group typing.

Preoperative documentation
The surgeon’s consultation note and the patient’s chart must include:

- Surgical approach: laparoscopy vs. laparotomy (for laparoscopy, specify the extraction site for the specimen).
- Anticipated need for vascular clamping.
- Estimated bleeding risk according to the planned resection (hepatectomy, lobectomy, segmentectomy; left/right; extended or not).
- Presence or absence of cirrhosis.

Depending on estimated bleeding risk and baseline hemoglobin, a RAI (indirect antiglobulin test) is ordered and two units of red blood cells are cross‑matched prior to admission.

**2. Intraoperative period**

Preoperative carbohydrate loading
Unless contraindicated (gastric emptying disorders, type 1 diabetes), all patients receive 400 mL of a carbohydrate‑containing beverage (e.g. apple juice, Oasis®, Ice Tea®, Coca‑Cola®) at 06:00 on the morning of surgery.

Monitoring and equipment

- Arterial catheter if hepatic clamping is planned or if comorbidities justify invasive monitoring.
- Single‑lumen central venous catheter for CVP monitoring if:
  - Laparotomy
  - High risk of conversion
  - Technically demanding laparoscopy with potential need for vasopressors (e.g. noradrenaline)
- Urinary catheter in case of laparotomy or complex laparoscopy; no catheter for straightforward laparoscopy. The urinary catheter is removed at the end of surgery unless specific indications persist (prostatic obstruction, hemodynamic instability, etc.).
- Nasogastric tube is not inserted routinely. If needed, it is ideally removed at the end of surgery after gastric aspiration.
- No systematic abdominal drainage. Should a drain be placed, clear postoperative management instructions are written in the orders.

Anesthesia protocol

- Preference for anesthetic agents with rapid kinetics.
- Induction: sufentanil dose kept ≤10 µg.
- Maintenance with volatile anesthetics (potential pharmacologic preconditioning).
- If remifentanil infusion is used, the dose should not exceed 0.1 µg·kg⁻¹·min⁻¹ or 3 ng·mL⁻¹ in TCI to limit the risk of opioid‑induced hyperalgesia.
- Magnesium sulfate (MgSO₄) 50–70 mg·kg⁻¹ over 15 minutes, administered as early as possible after induction to reinforce NMDA receptor blockade.
- Ketamine bolus 0.5 mg·kg⁻¹ after skin incision (anti‑hyperalgesic, analgesic and anti‑inflammatory effects).
- Clonidine (Catapressan®) is used cautiously because of potential intra‑ and postoperative hypotension in the context of potentially hemorrhagic surgery; if needed, the maximal dose is 150 µg.

Regional anesthesia / analgesia

- Thoraco‑abdominal plane (TAP) block plus intravenous lidocaine is preferred for both laparoscopy and laparotomy.
  - Laparotomy: subcostal TAP block with 2 × 20 mL levobupivacaine (Chirocaïne®) 0.375% + adrenaline 5 µg·mL⁻¹.
  - Laparoscopy: lateral posterior TAP with the same mixture.
- IV lidocaine infusion is used only for small‑volume hepatectomies: start 30–45 minutes after TAP block to limit systemic toxicity (bolus 1.5 mg·kg⁻¹ followed by 2 mg·kg⁻¹·h⁻¹ based on ideal body weight). Ketamine (50 mg per 50 mL lidocaine 2%) may be added depending on age and clinical context.
- Epidural analgesia is not recommended for laparotomy because of bleeding risk and potential postoperative coagulopathy after hepatectomy.

Blood conservation

- Tranexamic acid (Exacyl®) 15–20 mg·kg⁻¹ given before hepatic parenchymal transection in case of hepatectomy/lobectomy.

Neuromuscular blockade

- Adequate muscle relaxation is essential to provide optimal surgical conditions. Because the anesthetic technique aims to reduce end‑tidal volatile concentration, higher cumulative doses of neuromuscular blocker may be required. If residual block persists (TOF <3 responses) at the end of surgery, sugammadex (Bridion®) should be used when rocuronium was administered.

Intravenous fluid management

- Avoid both fluid overload (which increases hepatic bleeding) and hypovolemia (which favors postoperative renal impairment and compromises splanchnic perfusion). For laparotomy, a CVP ≤5 cmH₂O is recommended.
- Start with Plasmalyte® 2–4 mL·kg⁻¹·h⁻¹ after an initial 500 mL bolus in laparoscopy.
- Assess volume status using direct and indirect markers: arterial pressure, heart rate, end‑tidal CO₂, urine output (keeping in mind pneumoperitoneum effects), pulse pressure variation, lactate (noting that lactate may rise without hemodynamic instability due to reduced clearance during clamping), and, when indicated, advanced hemodynamic monitoring.

Ventilation

- Lung‑protective ventilation with tidal volume around 6 mL·kg⁻¹.
- Respiratory rate adjusted to avoid marked hypercapnia (PaCO₂ tolerated up to ~45 mmHg).
- No PEEP during parenchymal transection; recruitment maneuvers and PEEP applied after hepatectomy.

Prevention of hypothermia

- Systematic use of active warming (forced‑air warming blanket and warming mattress), with prewarming initiated at least 15 minutes before anesthesia.

Antiemetic prophylaxis

- Combination of droperidol 0.625 mg and ondansetron 4 mg at the end of surgery, guided by Apfel risk score.

Antibiotic prophylaxis

- Cefazolin 2 g IV 30 minutes before incision, with re‑dosing (1 g) if surgery lasts >4 hours or if >50% blood volume is lost. In case of serious penicillin allergy, clindamycin (Dalacin®) 600 mg is used. Regimens are adapted if a bilio‑digestive anastomosis is planned.

Thromboprophylaxis

- Intraoperative intermittent pneumatic compression (Kendall® boots). Low molecular weight heparin is started on the evening of postoperative day 0, unless intraoperative bleeding risk was high.

Corticosteroids

- Dexamethasone 20 mg or methylprednisolone (Solumedrol®) 125 mg, adjusted according to diabetic status.

Ischemic and pharmacologic preconditioning

- Ischemic preconditioning: 5‑minute clamp of the hepatic hilum to assess feasibility and tolerance of subsequent clamping.
- Pharmacologic preconditioning: 2–3 short “pulses” of sevoflurane at ~1 MAC before clamping, according to hemodynamic tolerance.

Thiamine

- Thiamine 500 mg (two 250 mg ampoules) before clamping and repeated in the immediate postoperative period.

**3. Postoperative period**

Urinary catheter

- Remove the urinary catheter upon leaving the operating room, unless specific indications justify prolonged use.

Postoperative analgesia

- Multimodal analgesia targeting a pain score ≤3:
  - Paracetamol up to 4 × 1 g·day⁻¹ for small hepatectomies, 2–3 × 1 g·day⁻¹ for major resections.
  - Tramadol (Contramal®) 100 mg then 200 mg over 24 hours for laparotomy, or oxycodone (Oxynorm®) in case of tramadol intolerance.
  - NSAIDs (e.g. ketorolac, Taradyl®) at the end of surgery if hemodynamic status is stable, hepatectomy is not major, and no contraindication exists (renal failure, severe cardiac disease, recent peptic ulcer). Typical dose 40–90 mg over 48 hours.
  - IV lidocaine infusion may be continued for 4–24 hours after small‑volume hepatectomy (1.0–1.33 mg·kg⁻¹·h⁻¹).

Laboratory monitoring

- Postoperative “type II” lab panel (full blood count, coagulation, renal function, electrolytes, proteins, liver function tests) on postoperative day 1, and subsequently according to clinical course and risk of hepatic insufficiency.

Intravenous fluids

- Avoid postoperative fluid overload; adjust IV fluids to daily requirements. If urine output remains low despite adequate fluid therapy, administer a loop diuretic (furosemide/Lasix® or bumetanide/Burinex®).

Nutritional support

- The patient is encouraged to resume oral intake on postoperative day 0. If no nausea/vomiting and the patient feels able, light solid food (e.g. toast with jam, cream dessert) is offered on the evening of surgery.
- On postoperative day 1, breakfast is allowed; if tolerated, regular meals are resumed and IV fluids are stopped.
- A more cautious refeeding schedule is applied after major hepatectomy or extensive mobilization of the stomach/duodenum, and this must be specified in postoperative orders.
- Before discharge, dietitian advice is provided in patients with preoperative malnutrition.

Early mobilization

- On day 0, the patient is mobilized in the recovery room and encouraged to get out of bed 2–3 hours after return to the ward, if clinical status allows. A message is sent to physiotherapy via OMNIMAIL “RAC” when the patient leaves the operating room.
- On postoperative day 1, the patient should sit in a chair in the morning and afternoon; walking in and outside the room is encouraged by the physiotherapist.

Protocol adherence

- Adherence to the protocol is monitored by ward nurses specifically engaged in the ERAS program. They receive the list of eligible patients and ensure protocol compliance and documentation.

High‑dependency / ICU admission

- Routine ICU stay is not required, except in cases of:
  - Complex surgery with high risk of liver failure
  - Hemodynamic instability
  - Major intraoperative bleeding
  - Significant comorbidities, etc.

Discharge criteria

- Adequate oral intake without significant nausea/vomiting.
- Satisfactory pain control with oral analgesics.
- Recovery parameters entered into the GRACE database.
- For patients undergoing a segmentectomy (1–3 segments) on a healthy liver via laparoscopy, who followed the ERAS protocol and meet all discharge criteria, same‑day discharge is possible.
